# The silent burden of sciatica in developing nations: charting a path to reform

**DOI:** 10.1097/MS9.0000000000004518

**Published:** 2025-12-11

**Authors:** Muddassir Khalid, Muhammad Abdullah

**Affiliations:** a Department of Surgery, Nishtar Medical University, Multan, Pakistan; b Department of Anatomy, Farrukh Mukhtar College of Nursing, Mukhtar A Sheikh Hospital, Multan, Pakistan

*Dear Editor,*

Although the term “sciatica” has been used indiscriminately to describe a variety of leg and back symptoms[1], it is actually a clinical illness that is characterized by pain that travels down the sciatic nerve from the buttock[2]. Muscle weakness, tingling, or numbness could accompany this pain[3]. In some instances, it may lead to a loss of bladder or bowel control[4]. From a medical standpoint, a herniated disc compressing Sciatic nerve is the most frequent cause of sciatica[5]. Spondylolisthesis, degenerative disc disease, and spinal stenosis are further contributory diseases. These disorders frequently result from underlying spinal abnormalities or age-related changes. But the onset of sciatica is also strongly associated with work and physical risk factors. The following factors are linked to increased risk: pregnancy, obesity, bad posture, prolonged sitting, and repetitive heavy lifting[6].

These physical risk factors are common in low-income nations like India & Pakistan. A sizable section of the populace works by hand, is not well-informed on ergonomics, or does not have access to healthcare for prevention and rehabilitation[7]. Because of financial limitations and a lack of adequate medical infrastructure, sciatica is disproportionately common but is nonetheless misdiagnosed and poorly treated[8]. In my experience as a medical intern in the Department of Neurosurgery, a recurring challenge with sciatica management in Pakistan is the delayed presentation of patients. The majority of people experience lower back pain for months at a time, initially turning to local therapies, over-the-counter analgesics, or even nonevidence-based procedures like cupping, acupuncture, or even dangerous and invasive procedures like stabbing[9].

Economic limitations, a lack of organized referral processes, and restricted access to imaging such as MRI are the main causes of the considerable delay in diagnosis and treatment. Many people arrive at emergency rooms without a previous medical evaluation, and they are frequently given high-dose painkillers to relieve their symptoms[10].

One of the structural challenges is the shortage of trained neurosurgeons in underdeveloped countries like Pakistan[11]. In many regions, neurosurgery remains an overlooked specialty, and even where specialists exist, they are often out of reach due to high costs or overwhelming demand. As a result, patients seek help from general practitioners or medically knowledgeable family members, who provide repeated opioids without a proper diagnosis, exacerbating their misery and causing preventable complications[12]. Another underserved and vulnerable group is pregnant women, where sciatica symptoms are frequently dismissed as normal due to cultural perceptions. In Pakistan’s patriarchal society, where female healthcare access is already limited, this neglect results in undiagnosed and mismanaged pain, further impacting quality of life[13].

Two approaches are required to move forward: targeted interventions at the healthcare provider and public health levels. First and foremost, it is critical that frontline healthcare professionals and general practitioners (GPs) receive the necessary training to recognise and treat sciatica. Workshops for continuing medical education (CME), online certification courses, and expert-led lectures on diagnosis, warning signs, and referral procedures can all help achieve this. Participation might be increased by offering incentives for such initiatives through medical councils or pharmaceutical corporations[14].

Second, it’s critical to expand access to neurosurgical consultation in underprivileged areas. This may entail tele-neurosurgery services, mobile spine camps, or teaching family doctors the fundamentals of neuro-assessment. At the local level, awareness is crucial[15]. Worsening of symptoms can be avoided via public service announcements in the media, instructional booklets in outpatient departments, and ergonomics and back care workshops in schools and workplaces. Specialised “Low Back Pain Desks” should be set up in hospitals where patients can be promptly evaluated, given priority for imaging (X-ray/MRI), and directed to the appropriate experts. Sciatica should be recognized, not normalized, and managed as a condition that demands urgent attention rather than delayed intervention. Sciatica is not just a symptom; it’s a call to systemic healthcare inequity. Early diagnosis, community education, and provider training can transform outcomes for millions in developing nations. The time to act is now.

TITAN Guidelines: This manuscript complies with TITAN Guidelines, 2025, declaring no use of AI (15).

## Data Availability

Not applicable.

## Peer and provenance statement

Not commissioned.
